# Health effects of milk consumption: phenome-wide Mendelian randomization study

**DOI:** 10.1186/s12916-022-02658-w

**Published:** 2022-11-23

**Authors:** Shuai Yuan, Jing Sun, Ying Lu, Fengzhe Xu, Doudou Li, Fangyuan Jiang, Zhongxiao Wan, Xue Li, Li-Qiang Qin, Susanna C. Larsson

**Affiliations:** 1 Department of Big Data in Health Science School of Public Health, Center of Clinical Big Data and Analytics of The Second Affiliated Hospital, Zhejiang University School of Medicine, Hangzhou, China; 2 Unit of Cardiovascular and Nutritional Epidemiology, Institute of Environmental Medicine, Karolinska Institutet, Stockholm, Sweden; 3 Key Laboratory of Growth Regulation and Translational Research of Zhejiang Province, School of Life Sciences, Westlake University, Hangzhou, China; 4 Westlake Intelligent Biomarker Discovery Lab, Westlake Laboratory of Life Sciences and Biomedicine, Hangzhou, China; 5 Department of Nutrition and Food Hygiene, School of Public Health, Soochow University, Suzhou, China; 6 Unit of Medical Epidemiology, Department of Surgical Sciences, Uppsala University, Uppsala, Sweden

**Keywords:** Milk, Health, Mendelian randomization, PheWAS, Systematic review

## Abstract

**Background:**

We performed phenome-wide Mendelian randomization analysis (MR-PheWAS), two-sample MR analysis, and systemic review to comprehensively explore the health effects of milk consumption in the European population.

**Methods:**

Rs4988235 located upstream of the *LCT* gene was used as the instrumental variable for milk consumption. MR-PheWAS analysis was conducted to map the association of genetically predicted milk consumption with 1081 phenotypes in the UK Biobank study (*n*=339,197). The associations identified in MR-PheWAS were examined by two-sample MR analysis using data from the FinnGen study (*n*=260,405) and international consortia. A systematic review of MR studies on milk consumption was further performed.

**Results:**

PheWAS and two-sample MR analyses found robust evidence in support of inverse associations of genetically predicted milk consumption with risk of cataract (odds ratio (OR) per 50 g/day increase in milk consumption, 0.89, 95% confidence interval (CI), 0.84–0.94; *p*=3.81×10^−5^), hypercholesterolemia (OR, 0.91, 95% CI 0.86–0.96; *p*=2.97×10^−4^), and anal and rectal polyps (OR, 0.85, 95% CI, 0.77–0.94; *p*=0.001). An inverse association for type 2 diabetes risk (OR, 0.92, 95% CI, 0.86–0.97; *p*=0.003) was observed in MR analysis based on genetic data with body mass index adjustment but not in the corresponding data without body mass index adjustment. The systematic review additionally found evidence that genetically predicted milk consumption was inversely associated with asthma, hay fever, multiple sclerosis, colorectal cancer, and Alzheimer’s disease, and positively associated with Parkinson’s disease, renal cell carcinoma, metabolic syndrome, overweight, and obesity.

**Conclusions:**

This study suggests several health effects of milk consumption in the European population.

**Supplementary Information:**

The online version contains supplementary material available at 10.1186/s12916-022-02658-w.

## Background

As major components of traditional Western diets, milk products that contain many essential nutrients may a play role in human health [1]. A substantial number of studies have examined the association between milk consumption and a wide range of health outcomes, like diabetes [2], cardiovascular disease [3–5], and certain cancers [6–8], albeit with inconclusive findings. The US dietary guideline recommends at least three servings (237 ml) per day of milk or equivalent portions of cheese, yogurt, or other dairy products for adults and children aged ≥9 years [9]. This standard is substantially higher than the current mean milk consumption (around 1.6 serving/day) among American adults [1] and populations in other parts of the world [10]. However, whether health benefits can be observed for such increased levels of milk consumption to justify the current dietary intake recommendation remains uncertain according to a recent review that scarcely found any well-established associations between milk consumption and disease risk [1]. In addition, most evidence is based on observational studies that are prone to be influenced by methodological limitations, particularly confounding and reverse causality.

Milk consumption is substantially influenced by the lactase gene (*LCT*), which encodes the enzyme lactase that is essential for lactose digestion. A genetic variant located upstream of the *LCT* gene was associated with lactase persistence and higher milk consumption in individuals of European descent [11, 12]. Mendelian randomization (MR) is an epidemiological approach that can strengthen causal inference by using one or more genetic variants as instrumental variable for an exposure [13]. Previous MR studies found possible associations of genetically predicted milk consumption with obesity and metabolic syndrome [14–19], Alzheimer’s disease [20], multiple sclerosis [20], colorectal [21] and kidney [22] cancers, hay fever [23], and asthma [23]. However, not all potential milk-intake–related outcomes, such as cataract [24], have been examined by MR studies, and further studies are required to replicate and strengthen these findings. Phenome-wide association study (PheWAS) is featured as a hypothesis-free design with the integrality of well-defined and widely adopted phenome framework to generate associations for further examination. To comprehensively explore the health effects of milk consumption, we conducted this study to examine the associations of milk consumption with a wide range of diseases by conducting a PheWAS analysis in the UK Biobank study as well as a two-sample MR analysis where the *LCT* gene variant was used as proxy for milk consumption. Of note, the UK Biobank study is based on a generally healthy young population which may not be perfectly suitable to study certain outcomes with a low prevalence. Thus, we further conducted a systematic review of published MR studies on milk consumption to complement the findings.

## Methods

### Study design

**Fig. 1:**
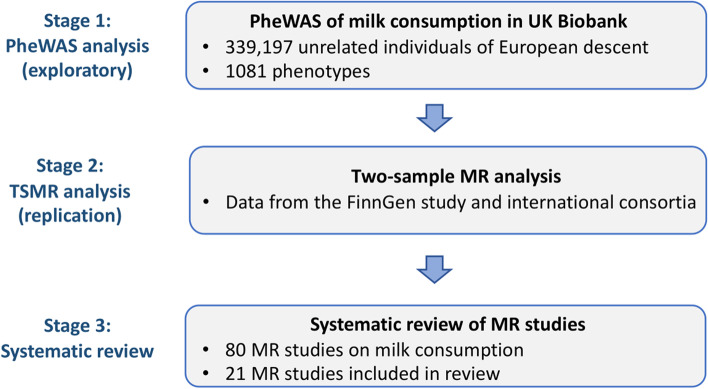
Study design overview. MR, Mendelian randomization; PheWAS, phenome-wide association analysis

Figure 1 shows the study design of the present investigation. We first conducted a MR-phenome-wide association study (MR-PheWAS) to explore the health effects of milk consumption in the UK Biobank study. We then conducted a two-sample MR analysis with data from the FinnGen study and international consortia to replicate the observed associations in the MR-PheWAS. Finally, we conducted a systematic review of MR studies on milk consumption to comprehensively synthesize the evidence to validate any possible health effects.

### Genetic instrument selection

A single nucleotide polymorphism (SNP, rs4988235) located upstream of the *LCT* gene was used as the instrumental variable in the MR-PheWAS and two-sample MR analyses. This SNP showed a strong association with milk consumption in the European populations [11]. One additional milk consumption increasing allele of rs4988235 was associated with an increase of 17.1 (95% confidence interval [CI] 10.6–23.6) g/day milk consumption in a sub-cohort of the European Prospective Investigation into Cancer and Nutrition-InterAct study including 12,722 participants [11]. Summary-level statistics on the rs4988235-milk association (i.e., beta and corresponding standard error coefficients) from the above study were used and rescaled to 50 g/day increase in the current MR analysis. The rs4988235-milk association was also verified in a cohort study comprising 73,715 Danish individuals where each additional milk consumption increasing allele of rs4988235 was associated with an increase of 0.58 (95% CI 0.49–0.68) glasses/week in milk consumption (*p*=9×10^−36^) [12]. In this study, rs4988235 explained around 2% phenotypic variance in milk consumption [12]. Rs4988235 explained around 2.5% variance in milk consumption and had a F-statistic of 1055 in the UK Biobank study. The milk consumption increasing allele of rs4988235 also showed a strong inverse association with lactose intolerance in the R6 FinnGen study data (*p*=1.54×10^−61^).

### MR-PheWAS in UK Biobank

We performed the PheWAS analysis using data on germline genotype and health outcomes from the UK Biobank study including 339,197 unrelated White individuals aged between 40 and 69 years in 2006–2010. The detailed quality control procedures of genotype data and the selection of white population are described in Additional file 1: Supplementary Methods and Fig. S1. Briefly, samples that were identified as a sex mismatch, outliers with high heterozygosity or with high missing rate, putative sex chromosome aneuploidy, individuals with excess relatives, or non-White ancestry were all excluded from the analysis. The largest possible subset of individuals without relatedness was identified using an algorithm implemented in the R package “*i-graph (v1.0.1)*” developed by Bycroft and colleagues [25]. The PheCODE schema [26] was used to define phenotypes based on an integrative application of 10,750 unique ICD (International Classification of Diseases)-10 codes and 3113 ICD-9 codes with diagnostic information from national medical records (e.g., inpatient hospital episode records, cancer registry, and death registry, Additional file 1: Table S1). Detailed information on genotyping and quality control in UK Biobank has been described in our previous studies [25, 27]. The UK Biobank received ethical permits from the North West Multi-centre Research Ethics Committee, the National Information Governance Board for Health and Social Care in England and Wales, and the Community Health Index Advisory Group in Scotland. All participants provided written informed consent.

### Two-sample MR

Two-sample MR analyses were conducted based on data from the R6 FinnGen study including up to 260,405 individuals [28] and international consortia [29–31]. The FinnGen study is a growing project combining data on germline genotypes and a wide range of health outcomes from Finnish biobanks and health registries. Detailed information on FinnGen and used international consortia is shown in Additional file 1: Table S2.

### Systematic review of MR studies on milk consumption

We further performed a systematic review of MR studies on milk consumption in the PubMed database to complement the associations obtained from the MR-PheWAS and two-sample MR analyses. We searched articles up to 3 March 2022 using the following search strategy: “Mendelian Randomization Analysis” [Mesh] OR mendelian[tiab] AND “milk” [Mesh] OR milk[tiab] (Additional file 1: Table S3). We extracted data on the first author, year of publication, used genetic instrument(s), outcome(s) studied, numbers of cases and controls, and the association estimates in the main statistical analysis. The literature search, review process, and data extraction were done in parallel by two authors (S.Y and Y.L.).

### Statistical analysis

In PheWAS, we calculated a weighted genetic score by adding up the number of milk consumption increasing alleles for rs4988235 weighted by its effect size on milk intake. We confined the analysis to outcomes with at least 200 cases [32]. The associations of genetically predicted milk consumption with phenotypes were estimated using logistic regression models adjusted for age, sex, body mass index (BMI), assessment center, and the first ten genetic principal components. We also conducted a sensitivity analysis without adjustment for BMI as well as additional stratification analyses of participants based on non-overweight (BMI <25 kg/m^2^) and overweight (BMI ≥25 kg/m^2^) status. The false discovery rate correction with the method by Benjamini-Hochberg was used to account for multiple comparisons in MR-PheWAS analysis [33].

In a two-sample MR analysis, the Wald ratio method was used to estimate the causal association (i.e., the beta coefficient for the effect of the SNP on the outcome divided by the beta coefficient for the effect of the SNP on milk consumption) [34]. The standard error of the ratio estimate is estimated using the delta method [35]. The odds ratio (OR) and corresponding CI were scaled to genetically predicted 50 g/day increase in milk consumption in MR-PheWAS and two-sample MR analyses. The association with a *p*<0.05 was deemed significant in the two-sample MR analysis. All tests were two-sided and conducted using a R package by Carroll et al [36], and MendelianRandomziation package [37] in R Software 4.0.2.

## Results

### MR-PheWAS analysis

**Fig. 2:**
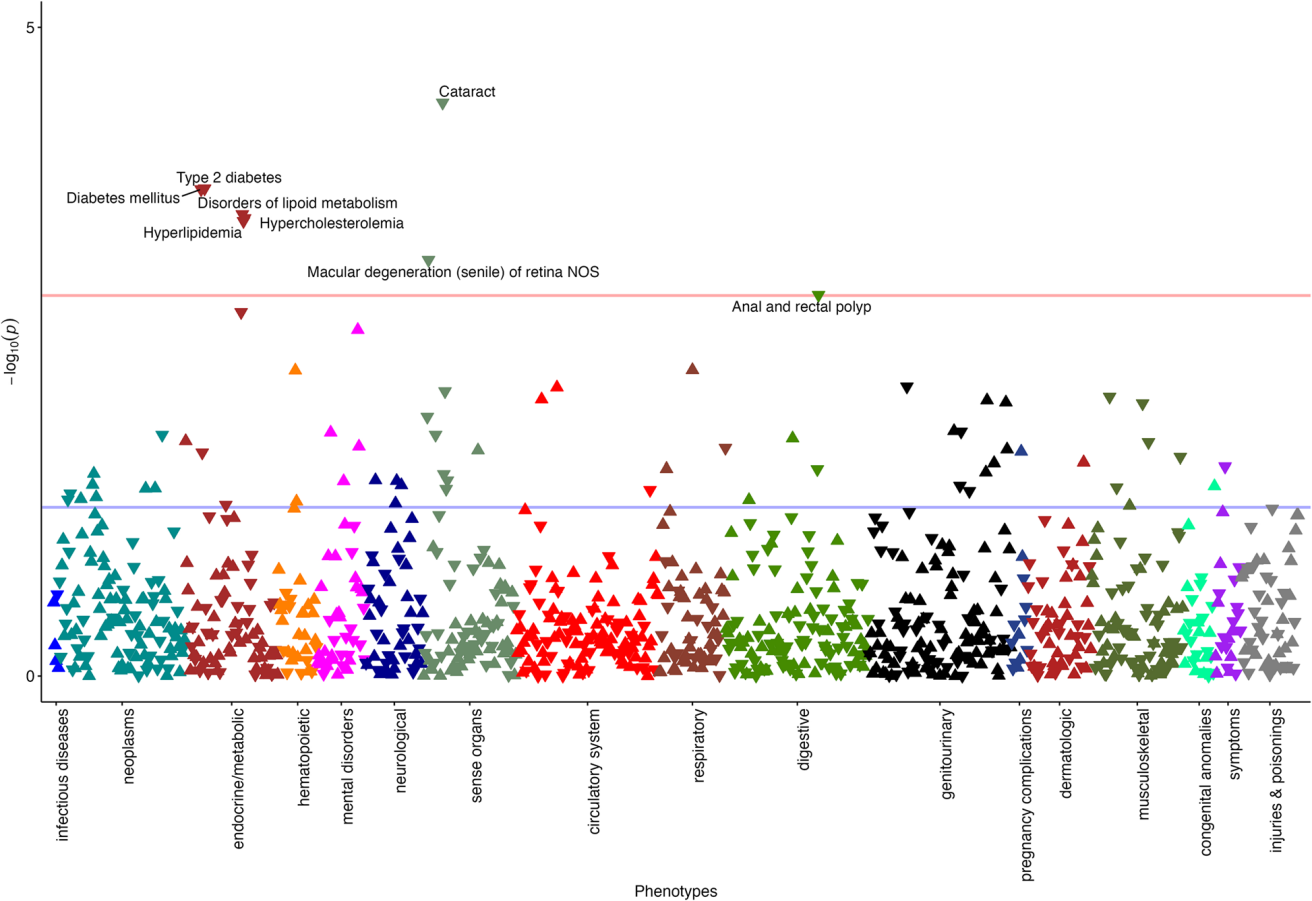
Results of the phenome-wide MR association analysis on genetically proxied milk consumption for clinical outcomes in the UK Biobank. The *Y*-axis corresponds to the logarithms of the *p* values derived from the phenome-wide MR association analyses. The red lines correspond to the statistical significance level (false discovery rate <0.05). Associations surviving the significance criteria are labeled by name

MR-PheWAS analysis was based on 182,072 females and 157,125 males in the UK biobank. The characteristics of participants are shown in Additional file 1: Table S4. Using the PheCODE schema, we defined 1853 distinct phenotypes. After the removal of outcomes with less than 200 cases, 1081 phenotypes classified into 18 disease categories were included in the analysis (Additional file 1: Table S5). A total of 70 phenotypes were associated with genetically predicted milk consumption at the nominal significance level (*p*<0.05) (Additional file 1: Table S6). After accounting for multiple testing, genetically predicted higher milk consumption was associated with decreased risk of 8 outcomes, including cataract, type 2 diabetes, diabetes mellitus, disorders of lipoid metabolism hypercholesterolemia, hyperlipidemia, macular degeneration (senile) of retina, and anal and rectal polyp (Table 1 and Fig. 2). The associations were stable in the sensitivity analysis without adjustment for BMI (Table 1). The associations were overall consistent in the analysis by overweight status albeit nonsignificant in the non-overweight population with small numbers of cases (Additional file 1: Table S7).

**Table 1:** Phenotypes associated with genetically proxied milk consumption in MR-PheWAS analysis in the UK Biobank (*N*=339,197)

Phecode	Phenotype	Cases	Controls	With adjustment for BMI				Without adjustment for BMI			
				Beta	SE	OR (95% CI)	***P***	Beta	SE	OR (95% CI)	***P***
366	Cataract	33,716	304,456	−0.120	0.029	0.89 (0.84–0.94)	3.81E−05	−0.114	0.029	0.89 (0.84–0.94)	8.63E−05
250.2	Type 2 diabetes	23,991	312,261	−0.129	0.035	0.88 (0.82–0.94)	1.75E−04	−0.094	0.032	0.91 (0.86–0.97)	4.36E−03
250	Diabetes mellitus	24,824	312,261	−0.126	0.035	0.88 (0.82–0.94)	1.78E−04	−0.091	0.032	0.91 (0.86–0.97)	4.49E−03
272	Disorders of lipoid metabolism	47,652	290,520	−0.094	0.026	0.91 (0.86–0.96)	2.77E−04	−0.076	0.026	0.93 (0.88–0.98)	2.10E−03
272.11	Hypercholesterolemia	43,956	290,520	−0.094	0.026	0.91 (0.86–0.96)	2.97E−04	−0.079	0.026	0.92 (0.88−0.97)	2.24E−03
272.1	Hyperlipidemia	47,448	290,520	−0.091	0.026	0.91 (0.87–0.96)	3.12E−04	−0.076	0.026	0.93 (0.88–0.98)	2.34E−03
362.29	Macular degeneration (senile) of retina	5276	321,852	−0.231	0.067	0.79 (0.70–0.91)	6.23E–04	−0.228	0.067	0.80 (0.70–0.91)	6.88E−04
565.1	Anal and rectal polyp	10,117	309,827	−0.161	0.050	0.85 (0.77–0.94)	1.16E−03	−0.155	0.050	0.86 (0.78–0.94)	1.65E−03

*BMI* body mass index, *CI* confidence interval, *OR* odds ratio, *SE* standard error. The associations were scaled to per 50 g/day increase in genetically predicted milk consumption

### Two-sample MR analysis

The associations for cataract, lipid metabolism, and anal and rectal polyp were observed in two-sample MR analysis (Table 2). Per 50 g/day increase in genetically predicted milk consumption, the OR was 0.97 (95% CI, 0.95, 0.99; *p*=0.006) for cataract, 0.91 (95% CI, 0.83, 0.98; *p*=0.015) for benign neoplasm of transverse colon, and 0.97 (95% CI, 0.94, 1.00; *p*=0.043) for benign neoplasm of colon. For the same increase in genetically predicted milk consumption, the changes of different lipid biomarkers were −0.015 (95% CI, −0.019, −0.012; *p*=1.97×10^−19^) for high-density lipoprotein cholesterol, −0.018 (95% CI, −0.022, −0.015; *p*=2.96×10^−27^) for low-density lipoprotein cholesterol, and −0.020 (95% CI, −0.023, −0.017; *p*=8.29×10^−34^) for triglycerides. Genetically predicted milk consumption was inversely associated with risk of type 2 diabetes in the analysis based on genome-wide association study data for type 2 diabetes with BMI adjustment (OR, 0.97, 95% CI, 0.96, 0.99; *p*=0.003), but not in the corresponding analysis based on data without BMI adjustment (Table 2).

**Table 2:** Associations of genetically proxied milk consumption with disease outcomes in two-sample Mendelian randomization analysis

Outcome	Source	Cases	Controls	Beta	SE	OR	95% CI	P
High-density lipoprotein cholesterol	GLGC	711,822	-	0.041	0.006	-	0.03–0.05	4.29E−12
Low-density lipoprotein cholesterol	GLGC	682,948	-	0.054	0.006	-	0.04–0.07	6.96E−20
Total cholesterol ^a^	GLGC	738,500	-	−0.004	0.006	-	−0.02–0.01	1.39E−22
Triglycerides	GLGC	699,618	-	0.057	0.006	-	0.05–0.07	0.468
Benign neoplasm of ascending colon	FinnGen	1605	208,187	−0.146	0.117	0.86	0.69–1.09	0.145
Benign neoplasm of transverse colon	FinnGen	1261	208,341	−0.292	0.117	0.75	0.59–0.94	0.015
Benign neoplasm of sigmoid colon	FinnGen	2695	257,710	−0.146	0.088	0.86	0.73–1.03	0.065
Benign neoplasm of colon	FinnGen	9208	203,426	−0.088	0.058	0.92	0.82–1.03	0.043
Benign neoplasm of colon, rectum, anus, and anal canal	FinnGen	11,490	202,006	−0.058	0.029	0.94	0.89–1.00	0.117
Type 2 diabetes	FinnGen	41,245	215,160	0.029	0.029	1.03	0.97–1.09	0.274
	DIAGRAM (no BMI adjustment)	74,124	824,006	−0.029	0.029	0.97	0.92–1.03	0.142
	DIAGRAM (BMI adjustment)	74,124	824,006	−0.088	0.029	0.92	0.86–0.97	0.003
Type 1 diabetes	Chiou J et al. GWAS	18,942	501,638	−0.058	0.058	0.94	0.84–1.06	0.184
Cataract	FinnGen	32,692	224,812	−0.088	0.029	0.92	0.86–0.97	0.006

*CI* confidence interval, *DIAGRAM* DIAbetes Genetics Replication And Meta-analysis, *GLGC* The Global Lipids Genetics Consortium, *OR* odds ratio, *SE* standard error. The associations were scaled to per 50 g/day increase in genetically predicted milk consumption

^a^ The levels were log-transformed

### Systematic review of MR studies on milk consumption

**Fig. 3:**
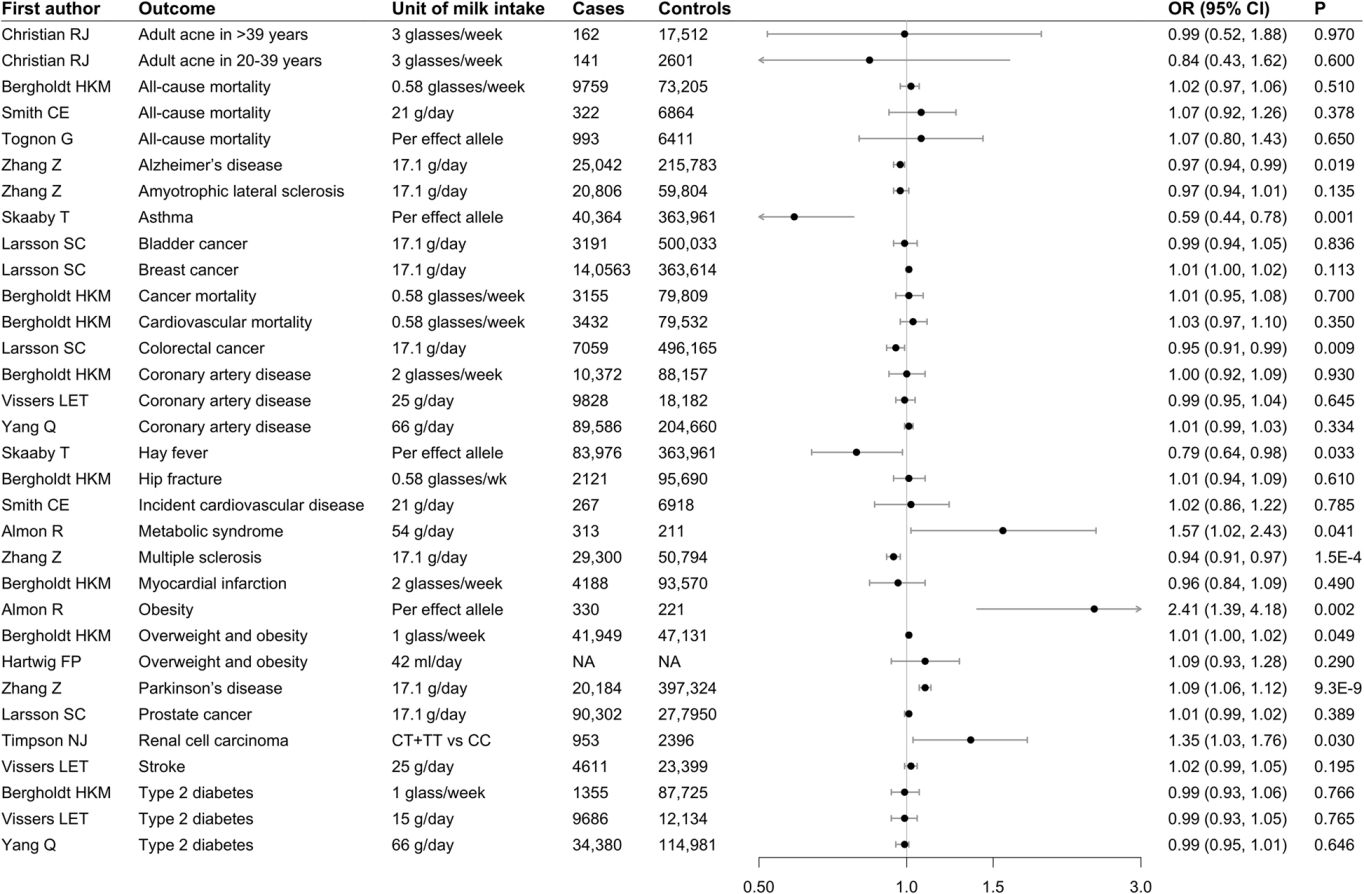
Associations of higher genetically proxied milk consumption with disease outcomes from a systematic review of Mendelian randomization studies. Estimates were obtained from the inverse-variance weighted method. CI, confidence interval; OR, odds ratio

A total of 80 studies were obtained from the search in the PubMed database and 18 relevant studies were included in the review. Information on 18 studies is present in Additional file 1: Table S8. For disease outcomes, genetically predicted higher milk consumption was associated with a lower risk of asthma, hay fever, multiple sclerosis, colorectal cancer, and Alzheimer’s disease, and a higher risk of Parkinson’s disease, renal cell carcinoma, metabolic syndrome, overweight and obesity (Fig. 3). For biomarkers, genetically predicted higher milk consumption was associated with lower levels high- and low-density lipoprotein cholesterol and higher levels of fasting insulin (Table 3).

**Table 3:** Associations of higher genetically proxied milk consumption with biomarkers from a systematic review of Mendelian randomization studies (inverse-variance weighted method)

First author	Unit	Outcome	***N***	Beta (95% CI)	***P***
Ricardo Almon	Per effect allele	Body mass index	221	0.960 (0.080, 1.850)	0.033
Hartwig FP	42 ml/day	Diastolic blood pressure	2780	0.270 (−0.450, 0.990)	0.470
Hartwig FP	42 ml/day	Systolic blood pressure	2780	0.430 (−0.500, 1.370)	0.360
Hartwig FP	42 ml/day	Body mass index	2780	0.440 (0.000, 0.880)	0.050
Yang Q	66 g/day	Low-density lipoprotein cholesterol	577	−0.028 (−0.036, −0.020)	2.30E−11
Yang Q	66 g/day	High-density lipoprotein cholesterol	577	−0.014 (−0.022, −0.006)	0.001
Yang Q	66 g/day	Femoral neck bone mineral density	53,236	−0.004 (−0.023, 0.015)	0.693
Yang Q	66 g/day	Fasting glucose	108,557	0.001 (−0.003, 0.005)	0.637
Yang Q	66 g/day	Triglycerides	577	0.003 (−0.005, 0.011)	0.472
Yang Q	66 g/day	Lumbar spine bone mineral density	53,236	0.004 (−0.019, 0.027)	0.746
Yang Q	66 g/day	Hemoglobin A1c	46,368	0.004 (−0.004, 0.012)	0.332
Yang Q	66 g/day	Beta cell function	46,186	0.007 (−0.001, 0.015)	0.086
Yang Q	66 g/day	Waist-to-hip ratio	210,222	0.008 (0.000, 0.016)	0.050
Yang Q	66 g/day	Insulin resistance	46,186	0.009 (0.001, 0.017)	0.027
Yang Q	66 g/day	Fasting insulin	108,557	0.012 (0.006, 0.018)	1.01E−4
Yang Q	66 g/day	Forearm bone mineral density	53,236	0.013 (−0.06, 0.033)	0.740
Yang Q	66 g/day	Body mass index	324,870	0.016 (0.010, 0.022)	0.000
Yang Q	66 g/day	2h glucose after drinking the glucose solution	42,854	0.021 (−0.003, 0.045)	0.086
Vimaleswaran KS	50 g/day	Total cholesterol	418,840	−0.080 (−0.120, −0.050)	1.90E−06
Vimaleswaran KS	50 g/day	Low-density lipoprotein cholesterol	418,840	−0.070 (−0.100, −0.040)	3.60E−06
Vimaleswaran KS	50 g/day	High-density lipoprotein cholesterol	418,840	−0.040 (−0.060, −0.020)	3.00E−05
Vimaleswaran KS	50 g/day	Triglycerides	418,840	0.000 (−0.010, 0.010)	0.970
Vimaleswaran KS	50 g/day	Hemoglobin A1c	418,840	0.000 (−0.010, 0.000)	0.300
Vimaleswaran KS	50 g/day	Waist circumference	418,840	0.010 (−0.020, 0.040)	0.500
Vimaleswaran KS	50 g/day	C-reactive protein	418,840	0.010 (−0.010, 0.020)	0.400
Vimaleswaran KS	50 g/day	Systolic blood pressure	418,840	0.020 (−0.180, 0.230)	0.800
Vimaleswaran KS	50 g/day	Body mass index	418,840	0.040 (0.020, 0.060)	3.60E−05
Vimaleswaran KS	50 g/day	Diastolic blood pressure	418,840	0.080 (−0.050, 0.200)	0.200

*CI* confidence interval

## Discussion

In this study, MR-PheWAS and two-sample MR analyses found robust evidence in support of inverse associations of genetically predicted milk consumption with risk of cataract, hyperlipidemia, and anal and rectal polyps. An inverse association between genetically predicted milk intake and type 2 diabetes risk was observed in MR analysis based on data with BMI adjustment, but not in the analysis without BMI adjustment. Systematic review of MR studies revealed additional inverse associations of genetically predicted milk consumption with asthma, hay fever, multiple sclerosis, colorectal cancer, Alzheimer’s disease, and blood lipid levels, but positive associations for Parkinson’s disease, renal cell carcinoma, metabolic syndrome, overweight and obesity, and levels of BMI and fasting insulin.

A novel finding of our study is the observed inverse association between milk consumption and risk of cataract. This association has been studied in only a few previous studies with conflicting findings [24]. Current daily milk intake was associated with a reduced risk of cataract extraction in a cross-sectional analysis including 5930 individuals from the US National Health and Nutrition Examination Survey [24]. However, the inverse association with incident cataract was not clearly observed in a cohort analysis of 5860 subjects from the PREvención con DIeta MEDiterránea Study [38]. Notably, the participants in this study were in high cardiovascular risk (e.g., with a high prevalence of diabetes, hypercholesterolemia, and hypertension) [38], which is different from the UK Biobank individuals with a generally good health status. Our recent MR study found causal associations of several cardiovascular risk factors with an increased risk of cataract [39]. In the PREvención con DIeta MEDiterránea Study, participants with at least one cardiovascular risk factor were at a high risk of cataract. Thus, the moderate protective effect of milk consumption on cataract might not be observed in this population.

Previous MR study reported a significant association between milk consumption and colorectal cancer risk [21]; however, this association did not pass multiple corrections in our PheWAS analysis (*P*=0.039). Instead, we found a significant association between milk consumption and the risk of anal and rectal polyps. Given that colorectal polyp is an important risk factor for colorectal cancer, the observed MR association between milk consumption and anal and rectal polyp partly supported the inverse association between milk consumption and colorectal cancer as reported by previous MR studies [6]. In a meta-analysis of 15 cohort studies with 11,733 incident colorectal cancer patients, higher consumptions of total dairy products and total milk were associated with a lower risk of colorectal cancer [6]. In a recent diet-wide association study for risk of colorectal cancer, one standard deviation increment increase in milk consumption was associated with 5% lower risk of incident colorectal cancer in 396,792 adults from the European Prospective Investigation into Cancer and Nutrition (EPIC) study [40]. In addition, by comparing results for different sites of colorectal polyp, our findings imply that high milk intake may exert more protective effects on transverse colon and possibly on sigmoid colon compared to other sites.

Evidence on the association between milk consumption and risk of type 2 diabetes is inconsistent between observational and MR studies. A review based on 12 meta-analyses found that most studies supported an inverse association between the consumption of total milk, in particular low-fat milk, and risk of incident type 2 diabetes [2]. Nevertheless, this inverse association was not observed in MR studies [11, 16, 18]. The MR analysis using rs4988235 as genetic instrument for milk consumption found no association between milk intake and diabetes risk (OR, 0.99: 95% CI, 0.93, 1.05) in 9686 diabetes cases and 12,134 controls from the EPIC study [11]. Our study found an association between milk consumption and risk of type 2 diabetes in data with BMI adjustment, but not in the analysis without adjustment for BMI. Considering BMI appears to be a collider factor as well as a mediator in the association between milk and type 2 diabetes, the adjustment for BMI was likely to bias the association. Thus, our findings along with previous MR studies [11, 16, 18], do not support a causal association between milk consumption and type 2 diabetes risk.

Several other health outcomes have been linked to milk consumption in previous MR studies. There were inverse associations for asthma [23], hay fever [23], multiple sclerosis [20], and Alzheimer’s disease [20], and positive associations for Parkinson’s disease [20], renal cell carcinoma [22], metabolic syndrome [14], and overweight and obesity [16]. However, these outcomes were not captured by our PheWAS analysis since our study might be lack of power to detect such associations, like for multiple sclerosis, Alzheimer’s disease, and Parkinson’s disease with a low prevalence or smaller number of cases in the UK biobank participants. Future studies with larger sample sizes and independent study populations are required to replicate and validate these reported MR findings. For biomarkers, genetically predicted higher milk intake was associated with lower levels of high- and low-density lipoprotein cholesterol [18] and higher levels of BMI [15, 17–19] and fasting insulin [18].

Several mechanisms may explain above identified associations. We observed that milk consumption favored the blood lipid profile, which can partly explain the inverse associations between milk consumption and outcomes with a high level of lipids as a risk factor, such as cataract [41]. In addition, calcium, riboflavin, vitamin D, phosphorous are rich in milk and these nutrients exert various health effects [42, 43]. A clinic-based study found that a high intake of riboflavin was associated with the decreased risk of several cataract-related endpoints [44]. Milk consumption has been associated with profiles of gut microbiome, which may mediate the associations of milk consumption and identified health issues. A genome-wide association study found that lactase gene locus was associated with *Bifidobacterium* abundance [45] and the colonization of *Bifidobacterium animalis* markedly reduces the polyp burden and possibly the risk of colorectal cancer [46]. Another study implied that *Bifidobacterium* possibly mediated the association between increased milk consumption and lower levels of triglycerides [47].

There are several strengths of this study, including a valid genetic instrument for milk consumption [11], a wide range of phenotypes studied, an independent replication analysis using data from other sources, and a comprehensive collection of findings from a systematic review. Several limitations need consideration when interpreting our results. Since the used genetic instrument is strongly associated with milk intake but not with intake of other dairy products, this study could only examine the health effects of total milk intake but not the effects of fermented milk products, such as cheese and yogurt. In addition, we could not differentiate the associations for intake of skimmed and unskimmed milk in this analysis based on summary level data. Our analysis might be challenged by horizontal pleiotropy since the genetic variant used as the proxy for milk intake might be associated with intake of other foods [11]. However, this horizontal pleiotropy should be minimal given the modest associations between rs4988235 and intake of a few other foods [11]. Possible nonlinear associations of milk intake with health outcomes could not be explored due to insufficient statistical power with only one genetic instrument explaining a modest proportion (about 2%) of the variance in milk consumption. We might have overlooked certain weak associations in MR-PheWAS analysis due to an inadequate power caused by a small phenotypic variance explained by the milk intake-associated SNP and a small number of cases in the UK Biobank. Even though an MR-PheWAS analysis based on a larger sample of a meta-analysis of UK Biobank and FinnGen may increase power, this approach cannot be conducted due to a lack of individual-level data in FinnGen. In addition, we could not compare the associations in different units of milk consumption even though it did not hinder the causal inference in these associations.

## Conclusions

In summary, this study revealed several health effects of milk consumption in the European population with evidence from a comprehensive investigation based on different study designs. These findings suggest that promoting milk intake may act as a dietary strategy for certain diseases’ prevention, such as for cataract and hyperlipidemia. Future clinical trials are needed to verify our results.

## Supplementary Information

**Additional file 1: Supplementary Methods**. **Table S1.** Mappings of ICD-10 and ICD-9 codes to the phenotypes identified by MR-PheWAS at the nominal significance level (*p*<0.05). **Table S2.** Information on the FinnGen study and international consortia. **Table S3.** Search strategy in the PubMed database. **TableS4.** Characteristics of participants in the UK Biobank (*N*=339,197). **Table S5.** Outcomes included in the analyses and outcomes excluded due to power (N<200 cases). **Table S6.** Phenotypes associated with genetically predicted milk consumption in MR-PheWAS at the nominal significance level (*p*<0.05) among unrelated white British sample (*N*=339,197). **Table S7.** Phenotypes associated with genetically proxied milk consumption by overweight status in MR-PheWAS analysis in the UK Biobank. **Table S8.** Information on included studies in review. **Figure S1.** Flow diagram of quality control procedures and the selection of target population.

## Data Availability

Data from UK Biobank can be obtained via application (https://www.ukbiobank.ac.uk/). The UK Biobank is an open-access resource and bona fide researchers can apply to use the UK Biobank dataset by registering and applying at http://ukbiobank.ac.uk/register-apply/. This research was conducted using the UK Biobank study under Application Number 66354. Data used in two-sample MR analysis and review of MR studies can be obtained by a reasonable request to the corresponding author. Codes for MR-PheWAS can be obtained in https://github.com/xueli157/xueli157/blob/main/PheWAS/PheWAS%20Function_R%20script.txt

## Abbreviations

BMI: Body mass index
CI: Confidence interval
ICD: International Classification of Diseases
MR: Mendelian randomization
MR-PheWAS: Phenome-wide Mendelian randomization analysis
OR: Odds ratio
SNP: Single nucleotide polymorphism
