# Supplementary material for: Health effects of milk consumption: phenome-wide Mendelian randomization study
Source: BMC Med. 2022 Nov 23;20:455. doi: 10.1186/s12916-022-02658-w (PMC9694907; doi:10.1186/s12916-022-02658-w)
Supplement: Supplementary file 1 — Additional file 1: Supplementary Methods. Table S1. Mappings of ICD-10 and ICD-9 codes to the phenotypes identified by MR-PheWAS at the nominal significance level (p<0.05). Table S2. Information on the FinnGen study and international consortia. Table S3. Search strategy in the PubMed database. TableS4. Characteristics of participants in the UK Biobank (N=339,197). Table S5. Outcomes included in the analyses and outcomes excluded due to power (N<200 cases). Table S6. Phenotypes associated with genetically predicted milk consumption in MR-PheWAS at the nominal significance level (p<0.05) among unrelated white British sample (N=339,197). Table S7. Phenotypes associated with genetically proxied milk consumption by overweight status in MR-PheWAS analysis in the UK Biobank. Table S8. Information on included studies in review. Figure S1. Flow diagram of quality control procedures and the selection of target population. [file 12916_2022_2658_MOESM1_ESM.docx]

Supporting information for

**Health effects of milk consumption: Phenome-wide Mendelian randomization analysis and systematic review**

*Shuai Yuan, Jing Sun, Ying Lu, Doudou Li, Fangyuan Jiang, Zhongxiao Wan, Xue Li, Li-Qiang Qin, Susanna C. Larsson*

Table of Contents

[Supplementary Methods 2](#_Toc118661857)

[Table S1. Mappings of ICD-10 and ICD-9 codes to the phenotypes identified by MR-PheWAS at the nominal significance level (p<0.05). 3](#_Toc118661858)

[Table S2. Information on the FinnGen study and international consortia 7](#_Toc118661859)

[Table S3. Search strategy in the PubMed database 9](#_Toc118661860)

[Table S4. Characteristics of participants in the UK Biobank (N=339,197). 10](#_Toc118661861)

[Table S5. Outcomes included in the analyses and outcomes excluded due to power (N<200 cases). 11](#_Toc118661862)

[Table S6. Phenotypes associated with genetically predicted milk consumption in MR-PheWAS at the nominal significance level (p<0.05) among unrelated white British sample (N=339,197). 12](#_Toc118661863)

[Table S7. Phenotypes associated with genetically proxied milk consumption by overweight status in MR-PheWAS analysis in the UK Biobank 14](#_Toc118661864)

[Table S8. Information on included studies in review 15](#_Toc118661865)

[Fig. S1. Flow diagram of quality control procedures and the selection of target population 17](#_Toc118661866)

# **Supplementary Methods**

PheWAS analysis was constrained to White individuals with high quality genotype data in order to minimize the influence of diverse population structure within UK Biobank. The metrics used to select the target study population were based on the data fields created for the genotype QC in UK Biobank. The detailed process for selecting target population is described below.

1. **Sex mismatch**

There are two data fields available to describe the sex of samples. Field one is the self-reported sex submitted by participants (coded by ***data field 31*** as “male” and “female”) and the other is the genetic sex (coded by ***data field 22001*** as “male” and “female”) inferred from the calling genotypes on the male-specific region of the Y chromosome and the non-pseudoautosomal region of the X chromosome. When self-reported sex was not consistent with the inferred sex from genotype data, samples were referred to as a sex mismatch. When comparing the ***data field 31*** with the ***data field 22001***, 99.9% samples showed concordance, but for a small number of samples (n=378) the data fields did not match and were thus excluded from the target population.

1. **Outliers in heterozygosity and missing rates**

The property of outliers in heterozygosity and high missing rates was coded as 0 (no) or 1 (yes) and was described in the variable: “*het.missing.outliers*”. A total of 968 samples (coded as 1 [“yes”]) were identified as outliers and were excluded from the study population.

1. **Putative aneuploidy in sex chromosome**

The property of putative aneuploidy in sex chromosome (putatively carrying sex chromosome configurations that are not either XX or XY) was coded as 0 (“no”) or 1 (“yes”) and was described in the variable: “*putative.sex.chromosome.aneuploidy*”. A total of 652 samples (coded as 1 [“yes”]) were identified as aneuploidy in sex chromosome and thus were excluded from the study population.

1. **Excess relatives**

The property of excess relatives (with more than 10 putative 3^rd^ degree relatives in the kinship table) was coded as 0 (“no”) or 1 (“yes”) and was described in the variable: “excess.relatives”. A total of 188 samples (coded as 1 [“yes”]) were identified to have excess relatives in UK Biobank and thus excluded from the study population.

1. **Non-White ancestry**

The property of British ancestry (self-reported ethnic background and genetic ethnic background as “White-British”) was coded as 0 (“no”) or 1 (“yes”). A total of 73,621 samples (coded as 0 [“no”]) did not belong to the White subset and thus were excluded from the study population.

1. **Unrelated samples**

The largest possible subset (vertices) of individuals without relatedness were identified using an algorithm implemented in the R package “i graph (v1.0.1)” developed by Bycroft et al.

By following the QC procedures, a total of 339,197 individuals restricted to the quality-filtered subset of White-British ancestry were included in the following analysis.

# **Table S1. Mappings of ICD-10 and ICD-9 codes to the phenotypes identified by MR-PheWAS at the nominal significance level (*p*<0.05).**

| **No** | **PheCODE** | **Phenotypes** | **Group** | **ICD-10** | **ICD-9** |
| --- | --- | --- | --- | --- | --- |
| 1 | 38 | Septicemia | infectious diseases | A02.1, A20.7, A22.7, A39.2-A39.4, A40-A40.3, A40.8-A41.5, A41.8, A41.9, A42.7, B00.7 | NA |
| 2 | 53 | Herpes zoster | infectious diseases | B02, B02.2, B02.7-B02.9 | NA |
| 3 | 153 | Colorectal cancer | neoplasms | C21.2, C21.8 | 154.8 |
| 4 | 153.3 | Malignant neoplasm of rectum, rectosigmoid junction, and anus | neoplasms | C19, C20, C21.0, C21.1, D01.1-D01.3 | 154, 154.0-154.3, 209.17, 230.4-230.6, 796.7, 796.70-796.74, 796.76, V10.06 |
| 5 | 165 | Cancer within the respiratory system | neoplasms | C38.4, C39, C39.0, C39.8, C39.9, C45.0, D02.1-D02.4, Z85.1, Z85.2, C33, C34-C34.3, C34.8, C34.9 | 162,162.2-163.1,163.8,163.9,165,165.8,165.9,209.21,231,231.1,231.2,231.8,231.9,V10.1,V10.11,V10.12,V10.2,V10.20,V10.29 |
| 6 | 174 | Breast cancer | neoplasms | C50-C50.9, D48.6, D05, D05.1, D05.7, D05.9, Z85.3 | 174,174.1-174.9,175,175.9,233,238.3,239.3,V10.3 |
| 7 | 174.1 | Breast cancer [female] | neoplasms | C50-C50.9, D48.6, D05, D05.1, D05.7, D05.9 | 174,174.1-174.9,233,V10.3 |
| 8 | 175 | Acquired absence of breast | neoplasms | Z90.1 | 612,612.1,V45.71,V51.0 |
| 9 | 202.24 | Large cell lymphoma | neoplasms | C83.3 | 200.6,200.61-200.68,200.7,200.71-200.78 |
| 10 | 210 | Benign neoplasm of lip, oral cavity, and pharynx | neoplasms | D10-D11.0, D11.7, D11.9 | 210,210.1-210.9 |
| 11 | 215 | Other benign neoplasm of connective and other soft tissue | neoplasms | D20.0, D20.1, D21, D21.0-D21.6, D21.9, D36.1 | 215, 215.0, 215.2-215.9 |
| 12 | 241 | Nontoxic nodular goiter | endocrine/metabolic | E04.8 | 241, 241.9 |
| 13 | 250 | Diabetes mellitus | endocrine/metabolic | E10-E10.4, E10.6-E11.4, E11.6-E11.9, E12.3, E13, E13.1, E13.3-E13.9, E14.9, G59.0, G63.2, H36.0, R73.0, R73.9, R81, R82.4, Z96.4 | 250, 250.0, 250.1, 250.2, 250.3, 250.4, 250.5, 250.6, 250.7, 250.8, 250.9 |
| 14 | 250.1 | Type 1 diabetes | endocrine/metabolic | E10-E10.4, E10.6-E10.9 | 250.01,250.03,250.11,250.13,250.21,250.23,,250.31,250.33,250.41,250.43,250.51,250.53,250.61,250.63,250.71,250.73,250.81,250.83,250.91,250.93 |
| 15 | 250.2 | Type 2 diabetes | endocrine/metabolic | E11, E11.0, E11.6-E11.9, E13, E13.5-E13.9, E14.9 | 250.00, 250.02, 250.20, 250.22, 250.30, 250.32, 250.80, 250.82, 250.90, 250.92 |
| 16 | 257 | Testicular dysfunction | endocrine/metabolic | E29, E29.0, E29.8, E29.9 | 257, 257.2, 257.8, 257.9 |
| 17 | 271 | Disorders of carbohydrate transport and metabolism | endocrine/metabolic | E73-E73.1, E73.8-E74.4, E74.8, E74.9, E76.0-E76.3, E76.8, E76.9 | 271,271.1-271.4,271.8,271.9,277.5 |
| 18 | 272 | Disorders of lipoid metabolism | endocrine/metabolic | E78.0-E78.5, E78.9 | 272,272.1-272.4,272.9 |
| 19 | 272.1 | Hyperlipidemia | endocrine/metabolic | E78.4, E78.5 | 272,272.1-272.4 |
| 20 | 272.11 | Hypercholesterolemia | endocrine/metabolic | E78.0 | 272 |
| 21 | 285.2 | Anemia of chronic disease | hematopoietic | D63.8 | 285.2, 285.21,285.22,285.29,285.3 |
| 22 | 285.22 | Anemia in neoplastic disease | hematopoietic | D63.0 | 285.22, 285.3 |
| 23 | 293 | Symptoms involving head and neck | mental disorders | R06.5, R06.7, R19.6 | 784,784.2, 784.9, 784.99 |
| 24 | 300 | Anxiety, phobic and dissociative disorders | mental disorders | F06.4, F34.1, F40-F40.2, F40.8-41.3, F41.8-F42.2, F42.8-F43.1, F48.0, F48.8, F48.9, F99, F93.1, F93.2, R45.2, R45.5, R45.7 | 293.84,300,300.01,300.02,300.09,300.1,300.2,300.21-300.23,300.29,300.3-300.5,300.89,300.9,308,308.1-308.4,308.9,308.81,313,313.1,313.21,313.22,313.3,313.82,313.83,V11.4 |
| 25 | 315 | Developmental delays and disorders | mental disorders | F63.3, F82, F88, F89, R45.1 | 307.9, 315, 315.02,315.09,315.3,315.31,315.32,315.34,315.35,315.39,315.4, 315.5, 315.8, 315.9, 317,318,318.1,318.2,319,V40,V40.1 |
| 26 | 315.1 | Learning disorder | mental disorders | F81.0-F81.2, F81.8, F81.9 | 315,315.01,315.1,315.2,V40.0 |
| 27 | 331.9 | Cerebral degeneration, unspecified | neurological | G31.0, G31.9 | 331.9 |
| 28 | 345 | Epilepsy, recurrent seizures, convulsions | neurological | G40, G40.9, G41.8 | 345, 345.9, 345.90 |
| 29 | 345.1 | Epilepsy | neurological | G40.1, G40.8, G41, G41.0, G41.1, G41.9 | 345.0, 345.00, 345.01, 345.2, 345.3, 345.6, 345.60, 345.61, 345.7, 345.70, 345.71, 345.8, 345.80, 345.81, 345.91 |
| 30 | 348.2 | Cerebral edema and compression of brain | neurological | G93.2, G93.5, G93.6 | 348.2, 348.4, 348.5 |
| 31 | 362 | Other retinal disorders | sense organs | H35, H35.8, H35.9 | 362, 362.8, 362.82, 362.85, 362.89, 362.9 |
| 32 | 362.2 | Degeneration of macula and posterior pole of retina | sense organs | H35.3 | 362.5,362.51-362.57 |
| 33 | 362.29 | Macular degeneration (senile) of retina NOS | sense organs | H35.3 | 362.5 |
| 34 | 364 | Corneal opacity and other disorders of cornea | sense organs | H18, H18.0, H18.3, H18.7-H18.9 | 371, 371.0, 371.1, 371.10-371.16, 371.24, 371.3, 371.30-371.33, 371.1, 371.70-371.73, 371.8, 371.81, 371.82, 371.89, 371.9 |
| 35 | 366 | Cataract | sense organs | H26, H26.2-H26.4, H26.8, H26.9, H28.0-H28.2, Z96.1 | 366, 366.3, 366.30-366.34, 366.4, 366.41-366.46, 366.5, 366.50-366.53, 366.8, 366.9, 998.82, V43.1, V45.61 |
| 36 | 366.2 | Senile cataract | sense organs | H25, H25.0-H25.2, H25.8, H25.9 | 366.1, 366.10-366.19 |
| 37 | 367 | Disorders of refraction and accommodation; blindness and low vision | sense organs | H52, H52.6, H52.7, Z97.3 | 367, 367.8, 367.89, 367.9, V41.0 |
| 38 | 367.1 | Myopia | sense organs | H52.1 | 367.1 |
| 39 | 367.2 | Astigmatism | sense organs | H52.2 | 367.2, 367.20-367.22 |
| 40 | 378.2 | Nystagmus and other irregular eye movements | sense organs | H55 | 379.5, 379.50-379.59 |
| 41 | 411.41 | Aneurysm and dissection of heart | circulatory system | I25.3, I25.4, I34.1 | 414.1, 414.11, 414.12, 414.19 |
| 42 | 420.21 | Acute pericarditis | circulatory system | I01.0, I30, I30.0, I30.1, I30.8, I30.9, I32 | 391.0, 420, 420.0, 420.9, 420.90, 420.91, 420.99 |
| 43 | 455 | Hemorrhoids | circulatory system | I84-I84.9 | 455, 455.1-455.9 |
| 44 | 473 | Diseases of the larynx and vocal cords | respiratory | J38.0-J38.7, J37-J37.1, R49-R49.2, R49.8 | 476, 476.1, 478.3, 478.31-478.34, 478.4-478.7, 478.71, 478.74, 478.75, 478.79, 784.4, 784.41-784.44, 784.49, V41.4 |
| 45 | 497 | Bronchitis | respiratory | J40 | 490 |
| 46 | 519.9 | Symptoms involving respiratory system and other chest symptoms | respiratory | R22.2 | 786, 786.6, 786.9 |
| 47 | 528.7 | Sialolithiasis | digestive | K11.5 | 527.5 |
| 48 | 550.5 | Ventral hernia | digestive | K43, K43.1, K43.9, K46.9 | 551.2, 551.20, 551.29, 552.2, 552.20, 552.29, 553.2, 553.20, 553.29 |
| 49 | 565 | Anal and rectal conditions | digestive | K59.4, K60-K60.5, K61-K61.4, K62.2-K62.4, K62.7-K62.9 | 564.6, 565, 565.0, 565.1, 566, 566.0, 569.1, 569.2, 569.4, 569.42-569.44, 569.49 |
| 50 | 565.1 | Anal and rectal polyp | digestive | K62.0, K62.1 | 569 |
| 51 | 594.3 | Calculus of ureter | genitourinary | N20.1 | 592.1 |
| 52 | 613.1 | Inflammatory disease of breast | genitourinary | N61, N64.0, N64.1 | 611, 611.2, 611.3 |
| 53 | 614.3 | Pelvic inflammatory disease (PID) | genitourinary | N70, N70.1, N70.9, N73.0-N73.2, N73.5, N73.9, N74.4 | 614, 614.1-614.4, 614.9 |
| 54 | 614.32 | Chronic inflammatory pelvic disease | genitourinary | N70.1, N73.1, N73.2 | 614.1, 614.4 |
| 55 | 614.54 | Abscess or ulceration of vulva | genitourinary | N76.4, N76.6 | 616.4, 616.5, 616.50, 616.51 |
| 56 | 622 | Polyp of female genital organs | genitourinary | N84.2, N84.3 | 623.7, 624.6 |
| 57 | 622.1 | Polyp of corpus uteri | genitourinary | D84, D84.0, D84.8, D84.9, | 621 |
| 58 | 624.9 | stress incontinence, female | genitourinary | N39.3 | 625.6 |
| 59 | 627 | Menopausal and postmenopausal disorders | genitourinary | N95.3, N95.8, N95.9 | 627, 627.8, 627.9 |
| 60 | 627.1 | Postmenopausal bleeding | genitourinary | N95.0 | 627.1 |
| 61 | 642 | Hypertension complicating pregnancy, childbirth, and the puerperium | pregnancy complications | O10-O10.1, O10.4, O10.9, O13, O14.1, O14.9, O15-O15.2, O15.9, O16 | 642, 642.01-642.04, 642.1, 642.11-642.14, 642.2, 642.21-642.24, 642.3, 642.31-642.34, 642.4, 642.41-642.44, 642.5, 642.51-642.54, 642.6, 642.61-642.64, 642.7, 642.71-642.74, 642.9, 642.91-642.94 |
| 62 | 709.2 | Sicca syndrome | dermatologic |  | 710.2 |
| 63 | 721.8 | Other allied disorders of spine | musculoskeletal | M40.2, M43.2, M48.1, M48.2, M48.9, M49.8, M53.9 | 721.5, 721.6, 721.8 |
| 64 | 726 | Peripheral enthesopathies and allied syndromes | musculoskeletal | M75, M75.1, M75.2, M75.4, M75.5, M75.8, M75.9, M77.0, M77.1, S46.0 | 726, 726.10, 726.13, 726.19, 726.2, 726.31, 726.32 |
| 65 | 728.2 | Laxity of ligament or hypermobility syndrome | musculoskeletal | M24.2, M35.7 | 728.4, 728.5 |
| 66 | 735 | Acquired foot deformities | musculoskeletal | M21.6 | 735, 736.7, 736.70-736.76, 736.79 |
| 67 | 735.3 | Hallux valgus (Bunion) | musculoskeletal | M20.1 | 727.1, 735.0 |
| 68 | 743.21 | Pathologic fracture of vertebrae | musculoskeletal | M48.5 | 733.13, V54.27 |
| 69 | 759 | Other and unspecified congenital anomalies | congenital anomalies | Q85.1, Q85.8, Q85.9, Q87.2, Q87.3, Q87.5, Q87.8, Q89.0-Q89.4, Q89.7-Q89.9 | 759-759.9, 759.89, V13.6, V13.69 |
| 70 | 780 | Hypothermia/Chills | symptoms | R68.0, T68 | 780.64, 780.65, 991.6 |

ICD indicates International Classification of Diseases.

# **Table S2. Information on the FinnGen study and international consortia**

| **Outcomes** | **Source** | **Population** | **Cases** | **Controls** | **PMID** | **ICD codes** | **Adjustment** |
| --- | --- | --- | --- | --- | --- | --- | --- |
| HDLC | GLGC | European | 711,822 |  | **34887591** | - | The analysis was conducted separately in male and female with adjustment for age, age^2^, principal components of ancestry, and any necessary study-specific covariates. |
| LDLC | GLGC | European | 682,948 |  | **34887591** | - |  |
| TC | GLGC | European | 699,618 |  | **34887591** | - |  |
| TG | GLGC | European | 738,500 |  | **34887591** | - |  |
| Malignant neoplasm of colorectum | FinnGen | European | 4401 | 204,070 | - | ICD-8: 153, 1540, 1541; ICD-9:153, 1540, 154; ICD-10: C18, C19, C20 | Age, sex, 10 genetic principal components, and genotyping batch. |
| Benign neoplasm of ascending colon | FinnGen | European | 1605 | 208,187 | - | ICD-8: 21133; ICD-10: D12.2 |  |
| Benign neoplasm of transverse colon | FinnGen | European | 1261 | 208,341 | - | ICD-8: 21134; ICD-10: D12.3 |  |
| Benign neoplasm of sigmoid colon | FinnGen | European | 2695 | 257,710 | - | ICD-8: 21136; ICD-10: D12.5 |  |
| Benign neoplasm of colon | FinnGen | European | 9208 | 203,426 | - | ICD-8: 21132, 21131, 21133, 21134, 21135, 21136, 21139; ICD-10: D12.0, D12.1, D12.2, D12.3, D12.4, D12.5, D12.6 |  |
| Benign neoplasm of colon, rectum, anus, and anal canal | FinnGen | European | 11490 | 202,006 | - | ICD-8: 21132, 21131, 21133, 21134, 21135, 21136, 21139, 2114 (ICD-8 and -9); ICD-10: D12.0, D12.1, D12.2, D12.3, D12.4, D12.5, D12.6, D12.7, D12.8, D12.9 |  |
| Senile cataract | FinnGen | European | 32,692 | 224,812 | - | ICD-8: 37402; icd-9: 3661; ICD-10: H25 |  |
| Type 2 diabetes | FinnGen | European | 41,245 | 215,160 | - | ICD-9: 2500A, 2502A, 2501A, 2503A, 2504A, 2505A, 2506A,2507A, 2508A; ICD-10: E11, N08.39, H36.09, G59.0, G63.2, G73.0, G99.0, M14.2; ATC: A10B |  |
| Type 2 diabetes | DIAGRAM | European | 74,124 | 824,006 | 30297969 | STable 1 (30297969) | Sex, principal components, and study-specific covariates. |
| Type 1 diabetes | Chiou J et al GWAS | European | 18,942 | 501,638 | 34012112 | STable 1 (34012112) | Sex, principal components, and genotyping batch. |

DIAGRAM, DIAbetes Genetics Replication And Meta-analysis; GLGC, The Global Lipids Genetics Consortium; GWAS, genome-wide association study; HDLC, high-density lipoprotein cholesterol; LDLC, low-density lipoprotein cholesterol; OR, odds ratio; PMID, PubMed identifier; TC, total cholesterol; TG, triglycerides.

Note: The UK Biobank study and FinnGen R3 were included in the GWAS on type 1 diabetes but not in other used data sources. The endpoints from FinnGen were defined by International Classification of Diseases-8, 9 and -10 codes, medicine purchase information, and surgical data. Detailed diagnostic codes can be obtained in <https://r6.finngen.fi/> In FinnGen, individuals with ambiguous gender, high genotype missingness (>5%), excess heterozygosity (±4 SDs), and non-Finnish ancestry had been excluded. In GLGC, individuals with call rate <95%, with heterozygosity > median + 3 (interquartile range), ancestry outliers from principal component (PC) analysis within each ancestry group and variants deviating from Hardy–Weinberg equilibrium (HWE; P <1 × 10−6) or with variant call rate <98% were removed. In Chiou J et al GWAS, individuals with missing genotypes (missing >5%), sex mismatch with phenotype records, cryptic relatedness through identity-by-descent, and non-European ancestry were excluded. Detailed information on outcome definition can be found in original genome-wide association studies. These genetic studies did not excluded individuals with treatments. For lipids, individuals on cholesterol-lowering medication had their pre-medication levels approximated by dividing the LDL-C value by 0.7 and the TC value by 0.8.

# **Table S3. Search strategy in the PubMed database**

| **Search** | **PubMed Query – 3 March, 2022** | **Items found** |
| --- | --- | --- |
| #3 | #1 AND #2 | 80 |
| #2 | "Mendelian Randomization Analysis"[Mesh] OR mendelian[tiab] | 15,658 |
| #1 | "milk"[Mesh] OR milk[tiab] | 159,916 |

# **Table S4. Characteristics of participants in the UK Biobank (N=339,197).**

| **Characteristics** | **Mean (SD)/ N (%)** |
| --- | --- |
| N | 339,197 |
| Age, mean (SD) | 56.9 (8.0) |
| Female, N (%) | 182,072 (53.7) |
| BMI, kg/m^2^, mean (SD) | 27.4 (4.8) |
| Genotype (rs4988235) |  |
| AA | 193,055 (56.9) |
| AG | 125.487 (37.0) |
| GG | 20, 655 (6.1) |

# **Table S5. Outcomes included in the analyses and outcomes excluded due to power (N<200 cases).**

| **Diagnostic category** | **N Phenotypes** | | | **N cases** | | |
| --- | --- | --- | --- | --- | --- | --- |
|  | **Total** | **Excluded due to power** | **Finally included** | **Median** | **Minimum** | **Maximum** |
| Circulatory system | 145 | 24 | 121 | 1884 | 200 | 96779 |
| Congenital anomalies | 52 | 25 | 27 | 528 | 216 | 1634 |
| Dermatologic | 79 | 28 | 51 | 1042 | 202 | 8884 |
| Digestive | 144 | 23 | 121 | 2153 | 219 | 46601 |
| Endocrine/ Metabolic | 109 | 30 | 79 | 802 | 203 | 47652 |
| Genitourinary | 150 | 29 | 121 | 1509 | 200 | 21598 |
| Hematopoietic | 50 | 17 | 33 | 798 | 203 | 17972 |
| Infectious disease | 52 | 24 | 28 | 1251 | 245 | 12313 |
| Injuries/poisonings | 77 | 25 | 52 | 1006 | 203 | 25606 |
| Mental disorders | 62 | 21 | 41 | 1126 | 208 | 19366 |
| Musculoskeletal | 120 | 42 | 78 | 1113 | 205 | 55332 |
| Neoplasms | 127 | 20 | 107 | 1538 | 220 | 29647 |
| Neurological | 72 | 23 | 49 | 622 | 209 | 13230 |
| Pregnancy complications | 27 | 13 | 14 | 807 | 216 | 2401 |
| Respiratory | 71 | 14 | 57 | 2047 | 202 | 18440 |
| Sense organs | 105 | 25 | 80 | 943 | 206 | 33716 |
| Symptoms | 31 | 9 | 22 | 1196 | 211 | 21745 |

# **Table S6. Phenotypes associated with genetically predicted milk consumption in MR-PheWAS at the nominal significance level (*p*<0.05) among unrelated white British sample (N=339,197).**

| **No** | **Phecode** | **Phenotypes** | **Group** | **Cases** | **Controls** | **Beta** | **SE** | **P** |
| --- | --- | --- | --- | --- | --- | --- | --- | --- |
| 1 | 366 | Cataract | sense organs | 33716 | 304456 | -0.120 | 0.029 | 3.81E-05 |
| 2 | 250.2 | Type 2 diabetes | endocrine/metabolic | 23991 | 312261 | -0.129 | 0.035 | 1.75E-04 |
| 3 | 250 | Diabetes mellitus | endocrine/metabolic | 24824 | 312261 | -0.126 | 0.035 | 1.78E-04 |
| 4 | 272 | Disorders of lipoid metabolism | endocrine/metabolic | 47652 | 290520 | -0.094 | 0.026 | 2.77E-04 |
| 5 | 272.11 | Hypercholesterolemia | endocrine/metabolic | 43956 | 290520 | -0.094 | 0.026 | 2.97E-04 |
| 6 | 272.1 | Hyperlipidemia | endocrine/metabolic | 47448 | 290520 | -0.091 | 0.026 | 3.12E-04 |
| 7 | 362.29 | Macular degeneration (senile) of retina | sense organs | 5276 | 321852 | -0.231 | 0.067 | 6.23E-04 |
| 8 | 565.1 | Anal and rectal polyp | digestive | 10117 | 309827 | -0.161 | 0.050 | 1.16E-03 |
| 9 | 362.2 | Degeneration of macula and posterior pole of retina | sense organs | 5279 | 321852 | -0.228 | 0.067 | 7.00E-03 |
| 10 | 271 | Disorders of carbohydrate transport and metabolism | endocrine/metabolic | 203 | 337969 | -1.003 | 0.319 | 1.57E-03 |
| 11 | 315 | Develomental delays and disorders | mental disorders | 918 | 337051 | 0.520 | 0.170 | 2.15E-03 |
| 12 | 497 | Bronchitis | respiratory | 852 | 320815 | 0.503 | 0.175 | 4.39E-03 |
| 13 | 285.2 | Anemia of chronic disease | hematopoietic | 1057 | 310306 | 0.450 | 0.158 | 4.42E-03 |
| 14 | 594.3 | Calculus of ureter | genitourinary | 2714 | 329898 | -0.257 | 0.094 | 5.90E-03 |
| 15 | 420.21 | Acute pericarditis | circulatory system | 257 | 333067 | 0.918 | 0.333 | 5.98E-03 |
| 16 | 367 | Disorders of refraction and accommodation; blindness and low vision | sense organs | 6236 | 331936 | -0.170 | 0.061 | 6.41E-03 |
| 17 | 721.8 | Other allied disorders of spine | musculoskeletal | 541 | 327990 | -0.547 | 0.202 | 7.07E-03 |
| 18 | 411.41 | Aneurysm and dissection of heart | circulatory system | 277 | 298797 | 0.863 | 0.322 | 7.39E-03 |
| 19 | 622.1 | Polyp of corpus uteri | genitourinary | 7641 | 326454 | 0.155 | 0.058 | 7.52E-03 |
| 20 | 627 | Menopausal and postmenopausal disorders | genitourinary | 10279 | 310319 | 0.135 | 0.050 | 7.80E-03 |
| 21 | 735 | Acquired foot deformities | musculoskeletal | 10654 | 322744 | -0.129 | 0.047 | 7.95E-03 |
| 22 | 53 | Herpes zoster | infectious diseases | 641 | 331933 | 0.526 | 0.205 | 9.66E-03 |
| 23 | 362 | Other retinal disorders | sense organs | 7130 | 321852 | -0.149 | 0.058 | 1.01E-02 |
| 24 | 613.1 | Inflammatory disease of breast | genitourinary | 721 | 336199 | 0.480 | 0.193 | 1.30E-02 |
| 25 | 614.32 | Chronic inflammatory pelvic disease | genitourinary | 388 | 329338 | -0.591 | 0.237 | 1.31E-02 |
| 26 | 293 | Symptoms involving head and neck | mental disorders | 1929 | 335125 | 0.287 | 0.117 | 1.33E-02 |
| 27 | 364 | Corneal opacity and other disorders of cornea | sense organs | 1701 | 321858 | -0.287 | 0.117 | 1.39E-02 |
| 28 | 215 | Other benign neoplasm of connective and other soft tissue | neoplasms | 655 | 330967 | -0.453 | 0.184 | 1.39E-02 |
| 29 | 550.5 | Ventral hernia | digestive | 4066 | 295967 | 0.193 | 0.079 | 1.48E-02 |
| 30 | 241 | Nontoxic nodular goiter | endocrine/metabolic | 2044 | 315730 | 0.269 | 0.111 | 1.55E-02 |
| 31 | 735.3 | Hallux valgus (Bunion) | musculoskeletal | 6783 | 322744 | -0.143 | 0.058 | 1.58E-02 |
| 32 | 315.1 | Learning disorder | mental disorders | 208 | 337051 | 0.886 | 0.371 | 1.71E-02 |
| 33 | 519.9 | Symptoms involving respiratory system and other chest symptoms | respiratory | 371 | 323586 | -0.576 | 0.243 | 1.75E-02 |
| 34 | 627.1 | Postmenopausal bleeding | genitourinary | 8669 | 310319 | 0.129 | 0.056 | 1.80E-02 |
| 35 | 378.2 | Nystagmus and other irregular eye movements | sense organs | 348 | 330183 | 0.658 | 0.278 | 1.83E-02 |
| 36 | 642 | Hypertension complicating pregnancy, childbirth, and the puerperium | pregnancy complications | 922 | 337250 | 0.398 | 0.170 | 1.87E-02 |
| 37 | 250.1 | Type 1 diabetes | endocrine/metabolic | 2903 | 312261 | -0.211 | 0.091 | 1.90E-02 |
| 38 | 743.21 | Pathologic fracture of vertebrae | musculoskeletal | 729 | 336512 | -0.409 | 0.175 | 2.05E-02 |
| 39 | 709.2 | Sicca syndrome | dermatologic | 718 | 324551 | 0.436 | 0.190 | 2.26E-02 |
| 40 | 624.9 | stress incontinence, female | genitourinary | 5623 | 329724 | 0.152 | 0.067 | 2.29E-02 |
| 41 | 780 | Hypothermia/Chills | symptoms | 397 | 337775 | -0.532 | 0.237 | 2.43E-02 |
| 42 | 473 | Diseases of the larynx and vocal cords | respiratory | 2979 | 318422 | 0.208 | 0.094 | 2.53E-02 |
| 43 | 565 | Anal and rectal conditions | digestive | 18371 | 309827 | -0.082 | 0.038 | 2.55E-02 |
| 44 | 622 | Polyp of female genital organs | genitourinary | 10262 | 326454 | 0.111 | 0.050 | 2.69E-02 |
| 45 | 174.1 | Breast cancer [female] | neoplasms | 11208 | 310156 | 0.105 | 0.050 | 2.75E-02 |
| 46 | 366.2 | Senile cataract | sense organs | 13364 | 304456 | -0.096 | 0.044 | 2.78E-02 |
| 47 | 38 | Septicemia | infectious diseases | 8601 | 319707 | 0.120 | 0.056 | 2.81E-02 |
| 48 | 331.9 | Cerebral degeneration, unspecified | neurological | 928 | 321374 | 0.360 | 0.167 | 3.09E-02 |
| 49 | 367.2 | Astigmatism | sense organs | 2263 | 331936 | -0.222 | 0.102 | 3.11E-02 |
| 50 | 345.1 | Epilepsy | neurological | 738 | 321374 | 0.404 | 0.187 | 3.13E-02 |
| 51 | 300 | Anxiety, phobic and dissociative disorders | mental disorders | 13524 | 309629 | 0.094 | 0.044 | 3.14E-02 |
| 52 | 174 | Breast cancer | neoplasms | 11293 | 310156 | 0.102 | 0.047 | 3.35E-02 |
| 53 | 348.2 | Cerebral edema and compression of brain | neurological | 481 | 321374 | 0.491 | 0.231 | 3.37E-02 |
| 54 | 614.3 | Pelvic inflammatory disease (PID) | genitourinary | 878 | 329338 | -0.345 | 0.164 | 3.42E-02 |
| 55 | 759 | Other and unspecified congenital anomalies | congenital anomalies | 227 | 336858 | 0.737 | 0.348 | 3.43E-02 |
| 56 | 726 | Peripheral enthesopathies and allied syndromes | musculoskeletal | 16081 | 312377 | -0.082 | 0.041 | 3.52E-02 |
| 57 | 210 | Benign neoplasm of lip, oral cavity, and pharynx | neoplasms | 1104 | 335392 | 0.319 | 0.152 | 3.57E-02 |
| 58 | 202.24 | Large cell lymphoma | neoplasms | 1003 | 331839 | 0.336 | 0.161 | 3.58E-02 |
| 59 | 367.1 | Myopia | sense organs | 2387 | 331936 | -0.205 | 0.099 | 3.61E-02 |
| 60 | 455 | Hemorrhoids | circulatory system | 14057 | 309407 | -0.088 | 0.041 | 3.68E-02 |
| 61 | 614.54 | Abscess or ulceration of vulva | genitourinary | 270 | 329338 | -0.596 | 0.287 | 3.75E-02 |
| 62 | 153.3 | Malignant neoplasm of rectum, rectosigmoid junction, and anus | neoplasms | 3343 | 300800 | -0.173 | 0.085 | 3.92E-02 |
| 63 | 175 | Acquired absence of breast | neoplasms | 4293 | 309239 | 0.155 | 0.076 | 4.17E-02 |
| 64 | 165 | Cancer within the respiratory system | neoplasms | 4658 | 333464 | 0.149 | 0.073 | 4.31E-02 |
| 65 | 153 | Colorectal cancer | neoplasms | 7117 | 300800 | -0.117 | 0.058 | 4.37E-02 |
| 66 | 528.7 | Sialolithiasis | digestive | 327 | 333614 | 0.582 | 0.289 | 4.39E-02 |
| 67 | 285.22 | Anemia in neoplastic disease | hematopoietic | 575 | 310306 | 0.430 | 0.213 | 4.51E-02 |
| 68 | 345 | Epilepsy, recurrent seizures, convulsions | neurological | 4309 | 321374 | 0.152 | 0.076 | 4.66E-02 |
| 69 | 257 | Testicular dysfunction | endocrine/metabolic | 228 | 332624 | -0.614 | 0.310 | 4.79E-02 |
| 70 | 728.2 | Laxity of ligament or hypermobility syndrome | musculoskeletal | 519 | 312377 | 0.442 | 0.222 | 4.85E-02 |

SE, standard error.

# **Table S7. Phenotypes associated with genetically proxied milk consumption by overweight status in MR-PheWAS analysis in the UK Biobank**

| **Phecode** | **Phenotype** | **BMI <25 kg/m^2^** | | | | | **BMI ≥25 kg/m^2^** | | | | |
| --- | --- | --- | --- | --- | --- | --- | --- | --- | --- | --- | --- |
|  |  | **Cases** | **Controls** | **Beta** | **SE** | ***P*** | **Cases** | **Controls** | **Beta** | **SE** | ***P*** |
| 366 | Cataract | 9833 | 102,413 | -0.114 | 0.053 | 0.030 | 23,883 | 202,043 | -0.120 | 0.035 | 5.44E-04 |
| 250.2 | Type 2 diabetes | 2150 | 109,557 | -0.079 | 0.105 | 0.461 | 21,841 | 202,704 | -0.137 | 0.035 | 1.85E-04 |
| 250 | Diabetes mellitus | 2450 | 109,557 | -0.085 | 0.099 | 0.398 | 22,374 | 202,704 | -0.132 | 0.035 | 2.25E-04 |
| 272 | Disorders of lipoid metabolism | 9293 | 102,953 | -0.041 | 0.056 | 0.437 | 38,359 | 187,567 | -0.108 | 0.029 | 1.70E-04 |
| 272.11 | Hypercholesterolemia | 8544 | 102,953 | -0.029 | 0.056 | 0.586 | 35,412 | 187,567 | -0.114 | 0.029 | 1.18E-04 |
| 272.1 | Hyperlipidemia | 9238 | 102,953 | -0.044 | 0.056 | 0.418 | 38,210 | 187,567 | -0.105 | 0.029 | 2.09E-04 |
| 362.29 | Macular degeneration (senile) of retina | 1529 | 107,223 | -0.073 | 0.126 | 0.566 | 3747 | 214,629 | -0.292 | 0.079 | 2.28E-04 |
| 565.1 | Anal and rectal polyp | 586 | 111,321 | -0.512 | 0.193 | 0.008 | 7585 | 205,268 | -0.123 | 0.058 | 0.030 |

BMI, body mass index; CI, confidence interval; SE, standard error.

# **Table S8. Information on included studies in review**

| **PMID** | **Year** | **First author** | **SNP** | **Unit** | **Outcome** | **Source** | **Cases** | **Controls** | **OR** | **LB** | **UB** |
| --- | --- | --- | --- | --- | --- | --- | --- | --- | --- | --- | --- |
| 19844753 | 2009 | Almon R | rs4988235 | 54 g/day | Metabolic syndrome | Canary Islands Nutrition Survey | 313 | 211 | 1.57 | 1.02 | 2.43 |
| 20447925 | 2010 | Timpson NJ | rs4988235 | CT+TT vs CC | Renal cell carcinoma | 7 studies | 953 | 2396 | 1.35 | 1.03 | 1.76 |
| 22937140 | 2012 | Almon R | rs4988235 | Per effect allele | Obesity | Canary Islands Nutrition Survey | 330 | 221 | 2.41 | 1.39 | 4.18 |
| 26156736 | 2015 | Bergholdt HKM | rs4988235 | 1 glass/wk | Type 2 diabetes | CGPS/GESUS | 1355 | 87725 | 0.99 | 0.93 | 1.06 |
|  |  |  |  |  | Overweight and obesity | CGPS/GESUS | 41949 | 47131 | 1.01 | 1.00 | 1.02 |
| 26085675 | 2015 | [Bergholdt HKM](https://pubmed.ncbi.nlm.nih.gov/?size=200&term=Bergholdt+HK&cauthor_id=26085675) | rs4988235 | 2 glasses/wk | Coronary artery disease | CCHS/CGPS/GESUS | 10372 | 88157 | 1.00 | 0.92 | 1.09 |
|  |  |  |  |  | Myocardial infarction | CCHS/CGPS/GESUS | 4188 | 93570 | 0.96 | 0.84 | 1.09 |
| 27624874 | 2016 | [Smith CE](https://pubmed.ncbi.nlm.nih.gov/?size=200&term=Smith+CE&cauthor_id=27624874) | rs3754686 | 21 g/day | Incident cardiovascular disease | PREDIMED | 267 | 6918 | 1.02 | 0.86 | 1.22 |
|  |  |  |  |  | All-cause mortality | PREDIMED | 322 | 6864 | 1.07 | 0.92 | 1.26 |
| 27170764 | 2016 | [Hartwig FP](https://pubmed.ncbi.nlm.nih.gov/?size=200&term=Hartwig+FP&cauthor_id=27170764) | rs4988235 | 42 ml/day | Overweight and obesity | 1982 Pelotas (Brail) Birth Cohort |  | 2780 | 1.09 | 0.93 | 1.28 |
| 28490510 | 2017 | [Tognon G](https://pubmed.ncbi.nlm.nih.gov/?size=200&term=Tognon+G&cauthor_id=28490510) | rs4988235 |  | All-cause mortality | NSHDS | 993 | 6411 | 1.07 | 0.80 | 1.43 |
| 28225053 | 2017 | Yang Q | rs4988235 | 66 g/day | Coronary artery disease | CARDIoGRAMplusC4D Metabochip & 1000 Genomes | 89586 | 204660 | 1.01 | 0.99 | 1.03 |
|  |  |  |  |  | Type 2 diabetes | DIAGRAM | 34380 | 114981 | 0.98 | 0.95 | 1.01 |
| 30096803 | 2018 | [Juhl CR](https://pubmed.ncbi.nlm.nih.gov/?size=200&term=Juhl+CR&cauthor_id=30096803) | rs4988235 | 3 glasses/wk | Adult acne in 20-39 years | GESUS | 141 | 2601 | 0.84 | 0.43 | 1.62 |
|  |  |  |  |  | Adult acne in >39 years | GESUS | 162 | 17512 | 0.99 | 0.52 | 1.88 |
| 29071499 | 2018 | [Bergholdt HKM](https://pubmed.ncbi.nlm.nih.gov/?size=200&term=Bergholdt+HKM&cauthor_id=29071499) | rs4988235 | 0.58 glasses/wk | All-cause mortality | CCHS/CGPS | 9759 | 73205 | 1.02 | 0.97 | 1.06 |
|  |  |  |  |  | Cardiovascular mortality | CCHS/CGPS | 3432 | 79532 | 1.03 | 0.97 | 1.10 |
|  |  |  |  |  | Cancer mortality | CCHS/CGPS | 3155 | 79809 | 1.01 | 0.95 | 1.08 |
| 29537719 | 2018 | [Bergholdt HKM](https://pubmed.ncbi.nlm.nih.gov/?size=200&term=Bergholdt+HKM&cauthor_id=29537719) | rs4988235 | 0.58 glasses/wk | Hip fracture | CCHS/CGPS/GESUS | 2121 | 95690 | 1.01 | 0.94 | 1.09 |
| 30728219 | 2019 | [Vissers LET](https://pubmed.ncbi.nlm.nih.gov/?size=200&term=Vissers+LET&cauthor_id=30728219) | rs4988235 | 15 g/day | Type 2 diabetes | EPIC-InterAct | 9686 | 12134 | 0.99 | 0.93 | 1.05 |
| 33261611 | 2020 | [Larsson SC](https://pubmed.ncbi.nlm.nih.gov/?size=200&term=Larsson+SC&cauthor_id=33261611) | rs4988235 | 17.1 g/day | Colorectal cancer | UK Biobank/FinnGen | 7059 | 496165 | 0.95 | 0.91 | 0.99 |
|  |  |  |  |  | Bladder cancer | UK Biobank/FinnGen | 3191 | 500033 | 0.99 | 0.94 | 1.05 |
|  |  |  |  |  | Breast cancer | UK Biobank/FinnGen/BCAC | 140563 | 363614 | 1.01 | 1.00 | 1.02 |
|  |  |  |  |  | Prostate cancer | UK Biobank/FinnGen/PRACTICAL | 90302 | 277950 | 1.01 | 0.99 | 1.02 |
| 34670632 | 2021 | Vissers LET | rs4988235 | 25 g/day | Stroke | EPIC-CVD/EPIC-NL | 4611 | 23399 | 1.02 | 0.99 | 1.05 |
|  |  |  |  |  | Coronary artery disease | EPIC-CVD/EPIC-NL | 9828 | 18182 | 0.99 | 0.95 | 1.04 |
| 34445060 | 2021 | [Zhang Z](https://pubmed.ncbi.nlm.nih.gov/?size=200&term=Zhang+Z&cauthor_id=34445060) | rs4988235 | 17.1 g/day | Multiple sclerosis | MS GWAS/MS Immunochip | 29300 | 50794 | 0.94 | 0.91 | 0.97 |
|  |  |  |  |  | Alzheimer’s disease | IGAP/FinnGen | 25042 | 215783 | 0.97 | 0.94 | 0.99 |
|  |  |  |  |  | Parkinson’s disease | PDWBS/PDGene | 20184 | 397324 | 1.09 | 1.06 | 1.12 |
|  |  |  |  |  | Amyotrophic lateral sclerosis | ALS Gwas | 20806 | 59804 | 0.97 | 0.94 | 1.01 |
| 34978666 | 2022 | Skaaby T | rs4988235 | Per effect allele | Hay fever | UK Biobank | 83976 | 363961 | 0.79 | 0.64 | 0.98 |
|  |  |  |  |  | Asthma | UK Biobank | 40364 | 363961 | 0.59 | 0.44 | 0.78 |
| 22937140 | 2012 | Almon R | rs4988235 | Per effect allele | Body mass index | Canary Islands Nutrition Survey | 551 |  | 0.96 | 0.08 | 1.85 |
| 27170764 | 2016 | [Hartwig FP](https://pubmed.ncbi.nlm.nih.gov/?size=200&term=Hartwig+FP&cauthor_id=27170764) | rs4988235 | 42 ml/day | Body mass index | 1983 Pelotas (Brail) Birth Cohort | 2780 |  | 0.44 | 0.00 | 0.88 |
|  |  |  |  |  | Systolic blood pressure | 1984 Pelotas (Brail) Birth Cohort | 2780 |  | 0.43 | -0.50 | 1.37 |
|  |  |  |  |  | Diastolic blood pressure | 1985 Pelotas (Brail) Birth Cohort | 2780 |  | 0.27 | -0.45 | 0.99 |
| 28225053 | 2017 | Yang Q | rs4988235 | 66 g/day | Forearm bone mineral density | GEFOS | 53236 |  | 0.01 | -0.06 | 0.03 |
|  |  |  |  |  | Femoral neck bone mineral density | GEFOS | 53236 |  | 0.00 | -0.02 | 0.02 |
|  |  |  |  |  | Lumbar spine bone mineral density | GEFOS | 53236 |  | 0.00 | -0.02 | 0.03 |
|  |  |  |  |  | Body mass index | GIANT | 324870 |  | 0.02 | 0.01 | 0.02 |
|  |  |  |  |  | Waist-to-hip ratio | GIANT | 210222 |  | 0.01 | 0.00 | 0.02 |
|  |  |  |  |  | Low-density lipoprotein cholesterol | GLGC | 577 |  | 0.03 | -0.04 | -0.02 |
|  |  |  |  |  | High-density lipoprotein cholesterol | GLGC | 577 |  | 0.01 | -0.02 | -0.01 |
|  |  |  |  |  | Triglycerides | GLGC | 577 |  | 0.00 | -0.01 | 0.01 |
|  |  |  |  |  | Hemoglobin A1c | MAGIC | 46368 |  | 0.00 | -0.01 | 0.01 |
|  |  |  |  |  | Fasting insulin | MAGIC | 108557 |  | 0.01 | 0.01 | 0.02 |
|  |  |  |  |  | Fasting glucose | MAGIC | 108557 |  | 0.00 | -0.01 | 0.01 |
|  |  |  |  |  | 2h glucose after drinking the glucose solution | MAGIC | 42854 |  | 0.02 | -0.01 | 0.05 |
|  |  |  |  |  | Beta cell function | MAGIC | 46186 |  | 0.01 | -0.01 | 0.02 |
|  |  |  |  |  | Insulin resistance | MAGIC | 46186 |  | 0.01 | 0.01 | 0.02 |
| 34024907 | 2021 | [Vimaleswaran KS](https://pubmed.ncbi.nlm.nih.gov/?size=200&term=Vimaleswaran+KS&cauthor_id=34024907) | rs4988235 | 50 g | Body mass index | 1958BC/HRS/UK Biobank | 418840 |  | 0.04 | 0.02 | 0.06 |
|  |  |  |  |  | Waist circumference | 1959BC/HRS/UK Biobank | 418840 |  | 0.01 | -0.02 | 0.04 |
|  |  |  |  |  | Systolic blood pressure | 1959BC/HRS/UK Biobank | 418840 |  | 0.02 | -0.18 | 0.23 |
|  |  |  |  |  | Diastolic blood pressure | 1960BC/HRS/UK Biobank | 418840 |  | 0.08 | -0.05 | 0.20 |
|  |  |  |  |  | High-density lipoprotein cholesterol | 1961BC/HRS/UK Biobank | 418840 |  | 0.04 | -0.06 | -0.02 |
|  |  |  |  |  | Low-density lipoprotein cholesterol | 1962BC/HRS/UK Biobank | 418840 |  | 0.07 | -0.10 | -0.04 |
|  |  |  |  |  | Total cholesterol | 1963BC/HRS/UK Biobank | 418840 |  | 0.08 | -0.12 | -0.05 |
|  |  |  |  |  | Triglycerides | 1964BC/HRS/UK Biobank | 418840 |  | 0.00 | -0.01 | 0.01 |
|  |  |  |  |  | Hemoglobin A1c | 1965BC/HRS/UK Biobank | 418840 |  | 0.00 | -0.01 | 0.00 |
|  |  |  |  |  | C-reactive protein | 1966BC/HRS/UK Biobank | 418840 |  | 0.01 | -0.01 | 0.02 |

BCAC, Breast Cancer Association Consortium; CCHS, Copenhagen City Heart Study; CGPS, Copenhagen General Population Study; DIAGRAM, DIAbetes Genetics Replication And Meta-analysis; GEFOS, GEnetic Factors for OSteoporosis Consortium; GESUS; Danish General Suburban Population Study; GIANT, international Genetic Investigation of ANthropometric Traits; GLGC, Global Lipids Genetics Consortium; HRS, Health and Retirement Study; IGAP, International Genomics of Alzheimer's Project; LB, lower bound of 95% confidence interval; MAGIC, Meta-Analyses of Glucose and Insulin-related traits Consortium; NSHDS, Northern Sweden Health and Disease Study; OR, odds ratio; SNP, single nucleotide polymorphism; PDWBS, Web-Based Study of Parkinson's Disease; PMID, PubMed identifier; PREDIMED, The Prevención con Dieta Mediterránea; PRACTICAL, The Prostate Cancer Association Group to Investigate Cancer Associated Alterations in the Genome; UB, upper bound of 95% confidence interval.

# **Fig. S1. Flow diagram of quality control procedures and the selection of target population**

485,223 participants with high quality genotype data

**Exclusion:**

Sex mismatch (n=378)

Outliers for heterozygosity and missing rate(n=968)

Sex chromosome aneuploidy (n=652)

With excess relatives (n=188)

**UK Biobank(n=487,409)**

Acquired data from participants who provided consent and had available genotype data

**Exclusion:**

Non-white ancestry (n=73,621)

Relatedness (n=72,405)

339,197 unrelated White British individuals
